# Supplementary figures and images for: The invasive longhorn beetle Xylotrechus chinensis, pest of mulberries, in Europe: Study on its local spread and efficacy of abamectin control
Source: PLoS One. 2021 Jan 29;16(1):e0245527. doi: 10.1371/journal.pone.0245527 (PMC7845995; doi:10.1371/journal.pone.0245527)

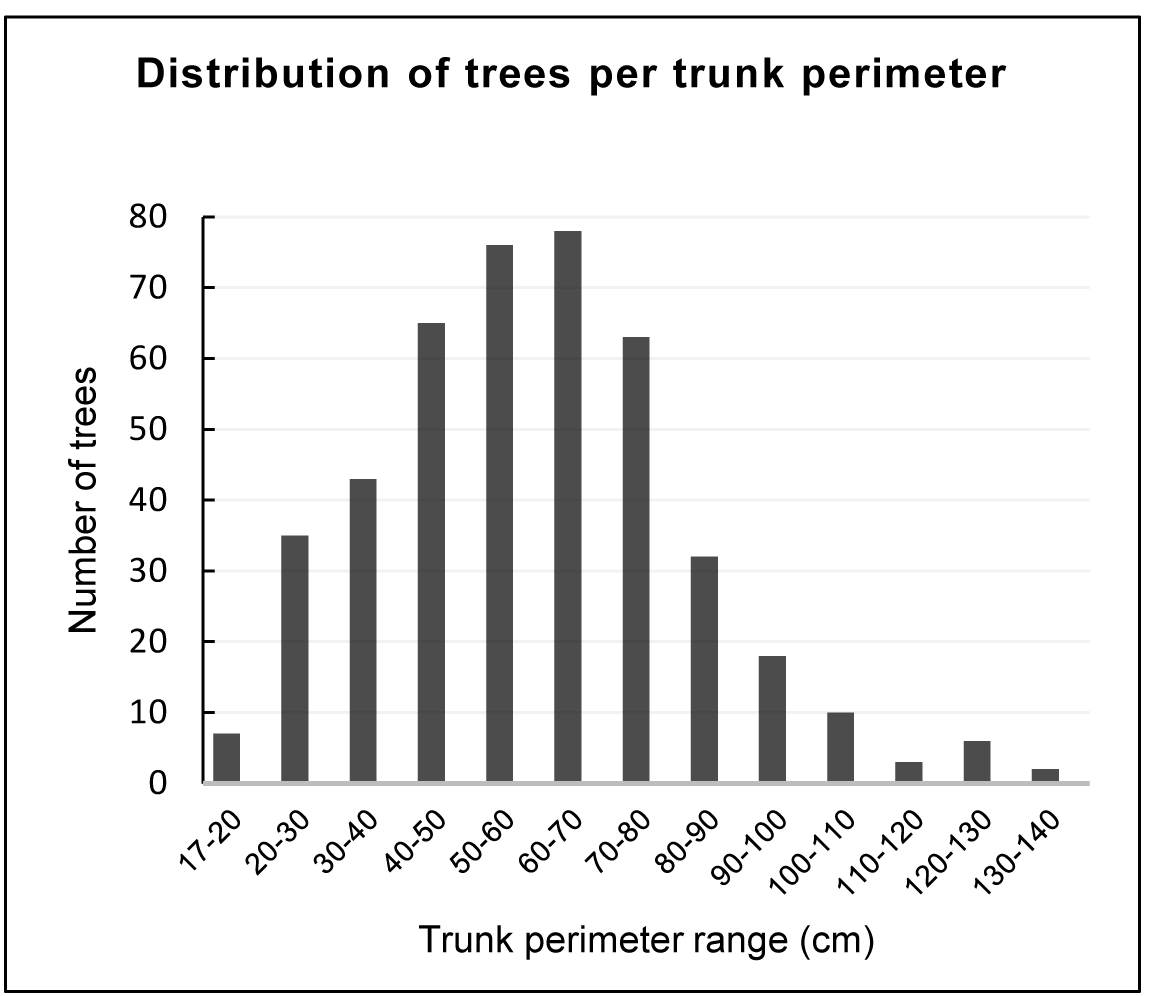

Supplement: S1 Fig — (TIF) [file pone.0245527.s001.tif]
