# Supplementary material for: The invasive longhorn beetle Xylotrechus chinensis, pest of mulberries, in Europe: Study on its local spread and efficacy of abamectin control
Source: PLoS One. 2021 Jan 29;16(1):e0245527. doi: 10.1371/journal.pone.0245527 (PMC7845995; doi:10.1371/journal.pone.0245527)
Supplement: S2 Fig — (PDF) [file pone.0245527.s002.pdf]

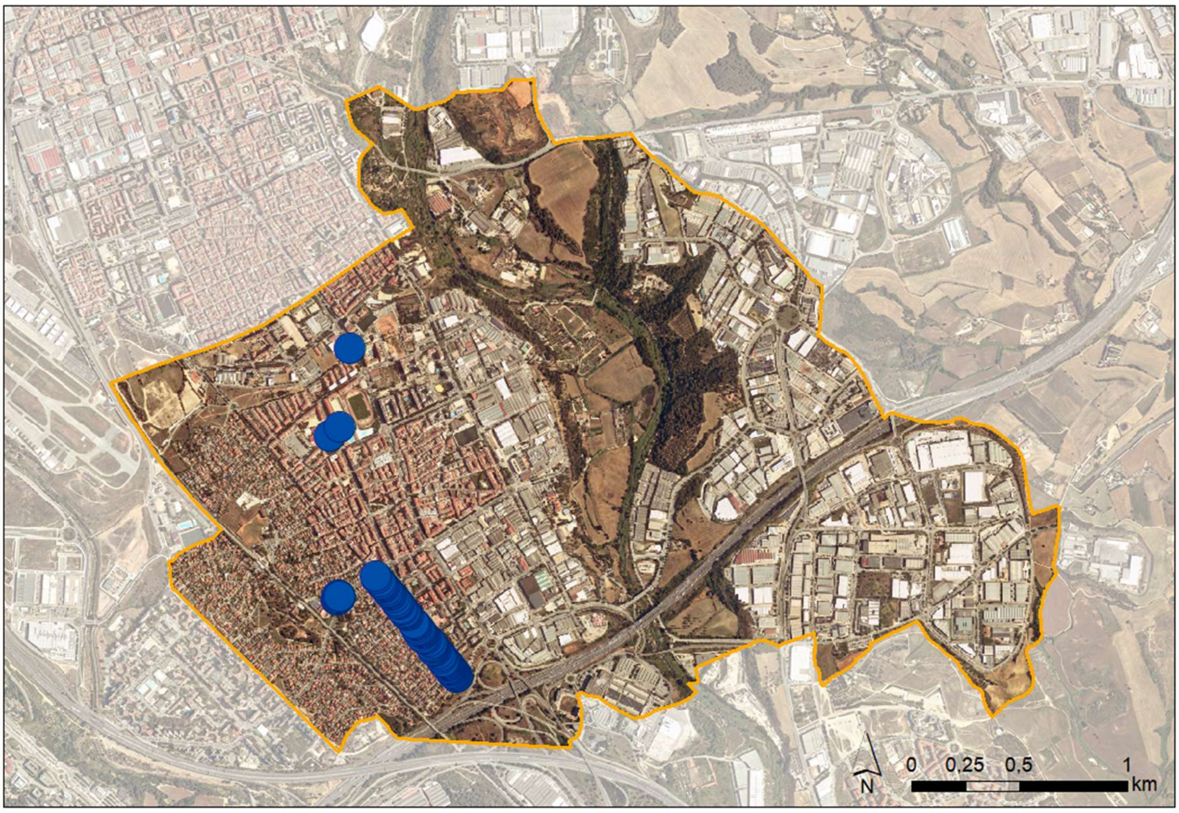

Location of the 99 trees treated with abamectin and selected by propensity score matching (below in more detail)

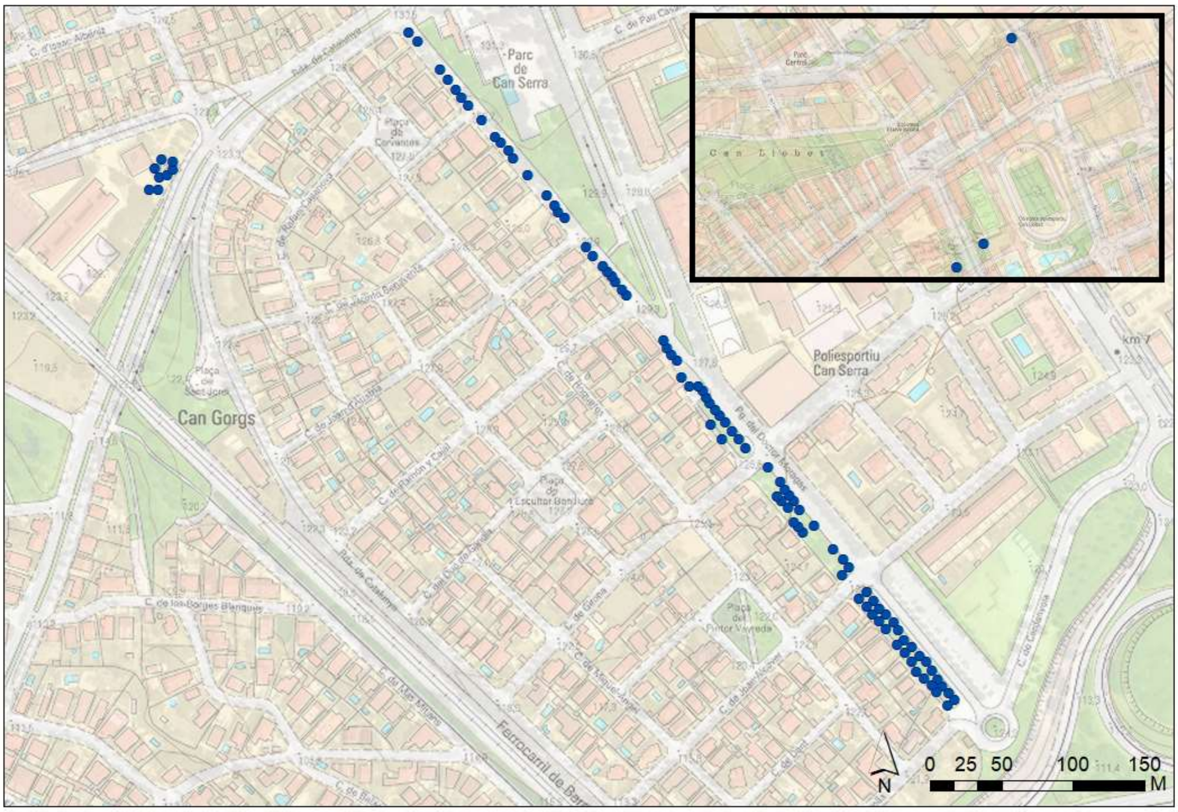

Note: Map sources from Cartographic and Geological Institute of Catalonia, https://www.icgc.cat/, under a CC BY 4.0 license, accessed 27 April 2020.

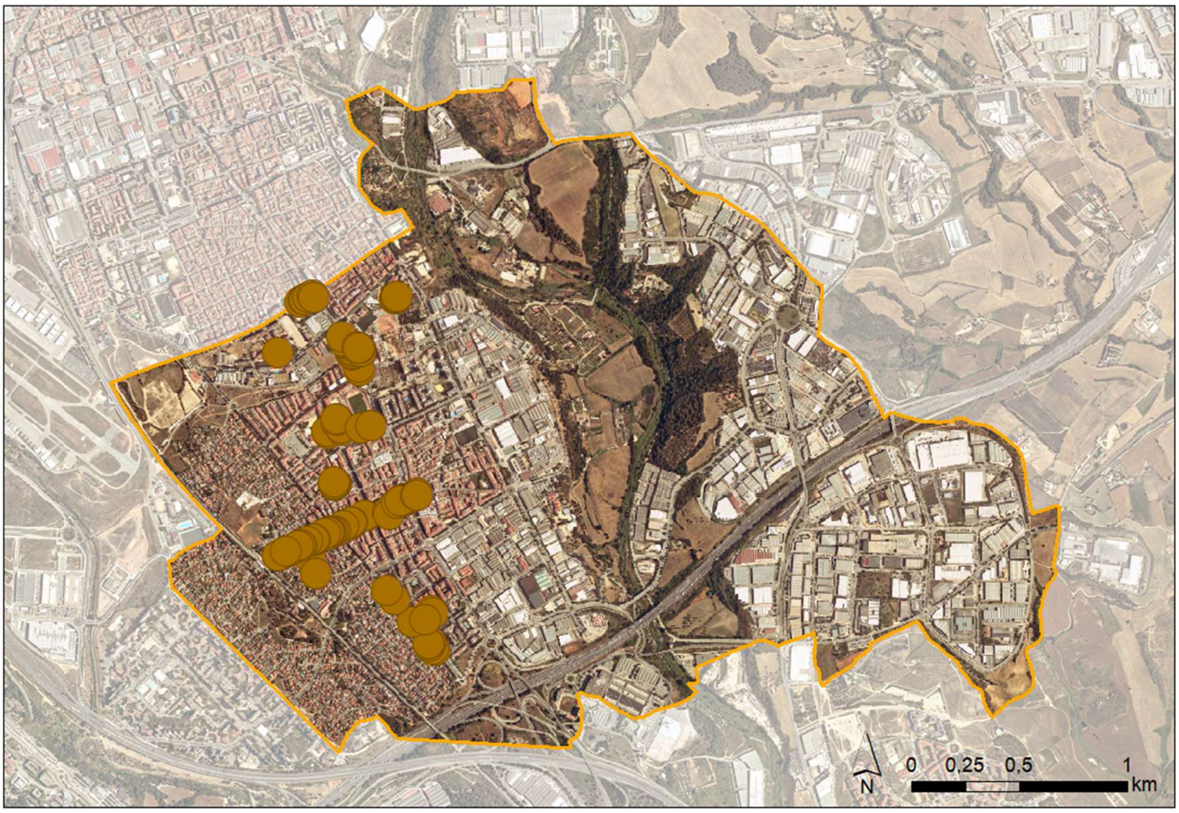

Location of the 99 trees selected as a control group by propensity score matching (below in more detail)

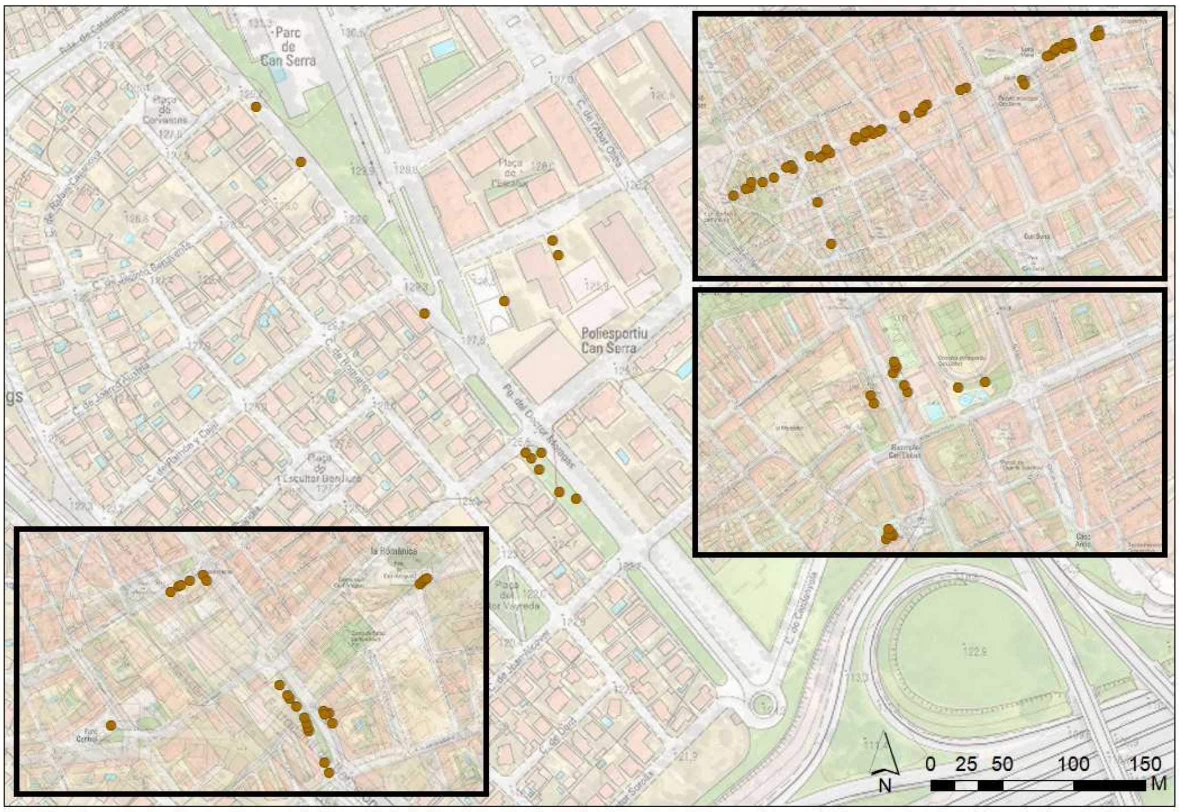

Note: Map sources from Cartographic and Geological Institute of Catalonia, https://www.icgc.cat/, under a CC BY 4.0 license, accessed 27 April 2020.
