# Supplementary material for: The invasive longhorn beetle Xylotrechus chinensis, pest of mulberries, in Europe: Study on its local spread and efficacy of abamectin control
Source: PLoS One. 2021 Jan 29;16(1):e0245527. doi: 10.1371/journal.pone.0245527 (PMC7845995; doi:10.1371/journal.pone.0245527)
Supplement: S4 Fig — (PDF) [file pone.0245527.s004.pdf]

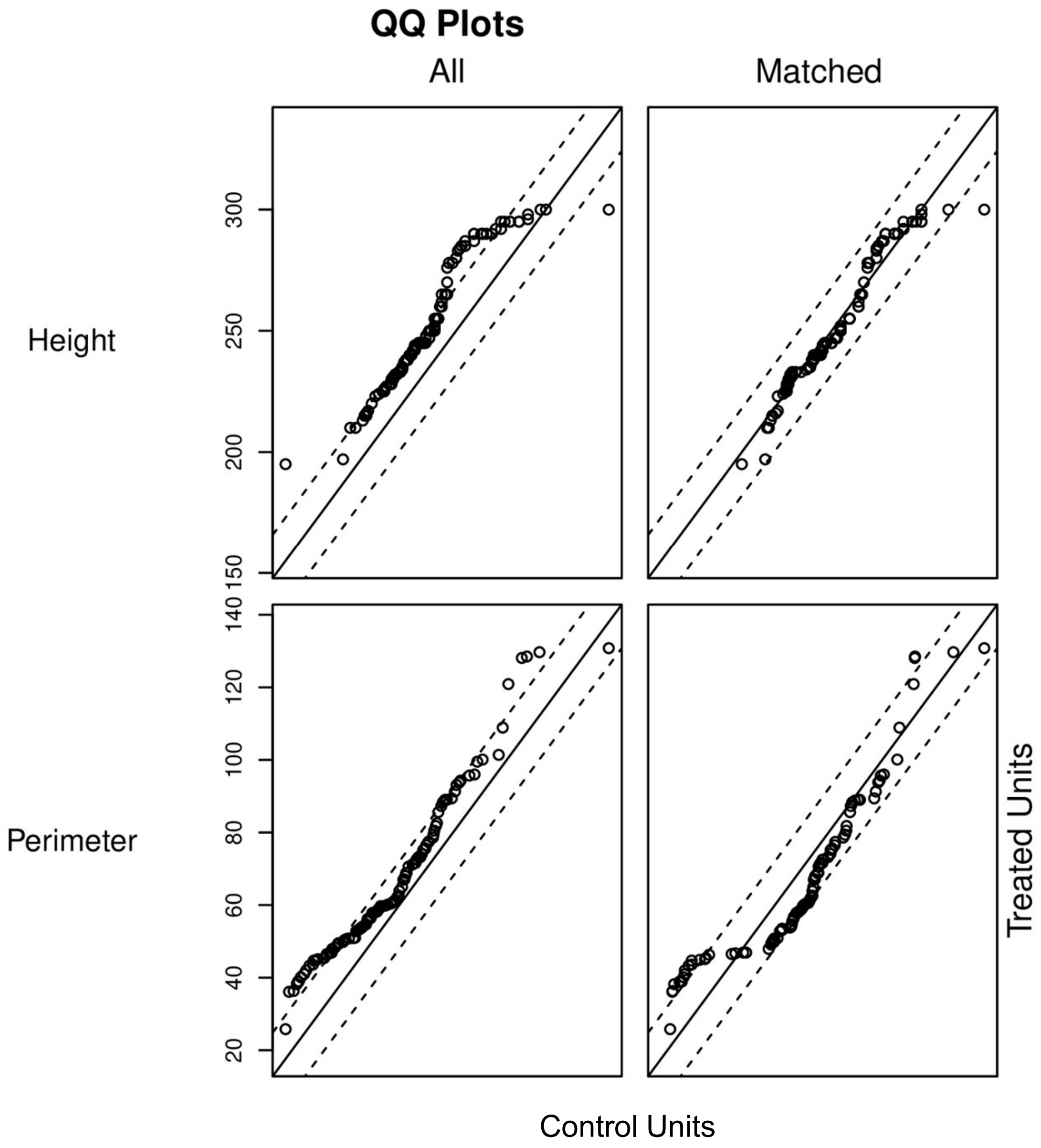

S4 Fig. QQ plots illustrating the matching for the two groups of observations as seen in S2 Table, for Height and Perimeter.
