# Supplementary material for: The invasive longhorn beetle Xylotrechus chinensis, pest of mulberries, in Europe: Study on its local spread and efficacy of abamectin control
Source: PLoS One. 2021 Jan 29;16(1):e0245527. doi: 10.1371/journal.pone.0245527 (PMC7845995; doi:10.1371/journal.pone.0245527)
Supplement: S2 Table — (PDF) [file pone.0245527.s006.pdf]

S2 Table. Comparison between treated and control groups of observations before and after applying propensity score matching.

	Before propensity score matching		After propensity score matching	
	Means treated	Means control	Means treated	Means control
EH_june	3.1308	0.4618	2.0404	0.9596
BI_june	0.5981	0.1993	0.5354	0.3939
GS_june	4.4019	0.9037	3.6061	2.1717
Height	252.82	235.87	252.89	253.73
Perimeter	66.77	57.52	65.83	69.21
N of obs	107	301	99	99

Clearly enough, after we match the two groups of observations, they become more similar on all the five characteristics under consideration.

*Here we list the codes of 99 trees treated with abamectin and selected by propensity score matching:
MA001 MA002 MA003 MA005 MA006 MA007 MA008 MA009 MA010 MA011 MA012 MA013 MA014 MA015 MA016 MA017 MA018 MA019 MA021 MA022 MA023 MA024 MA025 MA026 MA027 MA028 MA029 MA030 MA031 MA032 MA033 MA034 MA035 MA036 MA038 MA040 MA041 MA043 MA044 MA045 MA046 MA047 MA048 MA049 MA053 MA055 MA056 MA058 MA059 MA060 MA062 MA063 MA064 MA066 MA068 MA070 MA072 MA073 MA075 MA076 MA077 MA078 MA079 MA082 MA083 MA084 MA085 MA086 MA087 MA088 MA089 MA091 MA092 MA093 MA094 MA096 MA098 MA099 MA100 MA101 MA103 MA104 MA105 MA106 MA107 MA108 MA111 MA112 MA250 MA273 MA274 MA275 MA276 MA277 MA278 MA279 MA280 MA375 MA389

*And here we list the codes of 99 trees selected as a control group:
MA037 MA042 MA050 MA051 MA052 MA054 MA080 MA095 MA102 MA127 MA138 MA146 MA147 MA154 MA155 MA158 MA162 MA163 MA168 MA171 MA173 MA175 MA179 MA180 MA185 MA187 MA189 MA192 MA194 MA195 MA197 MA201 MA203 MA209 MA213 MA216 MA217 MA220 MA223 MA225 MA234 MA241 MA243 MA244 MA245 MA247 MA251 MA253 MA259 MA261 MA269 MA287 MA290 MA291 MA293 MA298 MA301 MA345 MA356 MA357 MA358 MA359 MA368 MA372 MA374 MA377 MA378 MA379 MA380 MA383 MA384 MA385 MA386 MA388 MA390 MA393 MA394 MA397 MA399 MA402 MA404 MA405 MA406 MA407 MA409 MA412 MA414 MA415 MA416 MA417 MA418 MA419 MA421 MA422 MA428 MA431 MA432 MA433 MA440
