# Supplementary material for: The invasive longhorn beetle Xylotrechus chinensis, pest of mulberries, in Europe: Study on its local spread and efficacy of abamectin control
Source: PLoS One. 2021 Jan 29;16(1):e0245527. doi: 10.1371/journal.pone.0245527 (PMC7845995; doi:10.1371/journal.pone.0245527)
Supplement: Attachment — Submitted filename: Response to Reviewers.docx [file pone.0245527.s008.docx]

# Responses to the Editor

# Title: "The invasive longhorn beetle *Xylotrechus chinensis*, pest of mulberries, in Europe: study on its local spread and efficacy of abamectin control”

# Manuscript ID: PONE-D-20-28834 *PLOS ONE*

# We do appreciate and are very grateful to the Editor for his good editing and valuable suggestions. Below we explain (in blue letters) how we have addressed such comments/suggestions.

Line 17 “a potentially lethal pest of mulberry trees (Moraceae: Morus sp.) **Done!**

Line 19 “the infestation spread” **Done!**

Line 22 “and avenues is a concern, as beetle infestation “ **Done!**

Line 23 “how the infestation progresses over time, with and without Abamectin treatment, and provide insights into female egg-laying preferences.  Such knowledge helps contribute to management efforts to reduce expansion of the range of beetle infestation.” **Done!**

Line 27 “do so on warmer, SW orientations rather than those facing N, NW and E. Emergence holes and gallery slits predict the spreading of infestations to new trees” **Done!**

Line 30 “begin feeding on the phloem” **Done!**

Line 45 Delete “As far as we know . . .described” **Done!**

Line 53 “This invasive species is native” **Done!**

Line 75 “into these French” **Done!**

Line 87 delete “, as we report here” **Done!**

Line96 “The data were assembled using ArcMap 10.3.1 . .” **Done!**

Line 112 “Spain and other southern European countries.” **Done!**

Line 114 Delete “Quite similar . . .countries.” **Done!**

Line 120 “concern because beetle infestation increases the risk of falling branches and the need for rapid response by municipal authorities.” **Done!**

Line 128 “observations, three types of indicators were selected to document infestation: (1) “ **Done!**

Line 130 “were counted and processed as described in the methods to inform the municipality about the progress of the beetle infestation in the study area.” **Done!**

Line 156 “Occasionally, full-grown larvae have been found overwintering under” **Done!**

Line 157 “Bark injuries become increasingly more evident “**Done!**

Line 160 “Consequently, the presence of bark injuries can be a good” **Done!**

Line 172 “which is bored into for pupation” **Done!**

Line 178 “No studies were reviewed that reported how” **Done!**

Line 180 “i.e., a statistical analysis of new infestation in relation to past infestation Nor could we find studies about the . . . perimeter, or about the geographical” **Done!**

Line 183 “provide important knowledge on the preferred trees for egg laying and where the eggs are most likely to be laid. A goal of the study was to determine such information to assist in scouting for infestations. **Done!**

Line 230 “Specifically, to aid in scouting, it is important to know” **Done!**

Line 234, or perhaps to wait until the discussion. Based on responses of other invasive beetle pests, one might also consider volatiles released by the trees, which, if were attractive would attract beetles over wide areas. The authors don’t bring this up, but many entomologists working in this research area would gravitate towards that possibility immediately. Potential attractants are not necessarily tree-based, so attractants of related longhorned beetles might be of interest.

**Authors’ response:** (Please, see also our response for Line 587 below). Indeed, what the Editor suggests is an important and interesting issue. Unfortunately, we did not work on that in our present work. However, we believe we should briefly mention these topics in our paper as something that should be studied in the future. Therefore, incorporating the Editor’s comments, we have added a brief paragraph at the end of the ‘Conclusions’ section that reads:

“*Finally, there are issues which have not been brought up in this work but may be important for the management of this pest. Indeed, based on responses of other invasive beetle pests, research is needed to detect volatiles released by mulberries, because if they were attractive would attract beetles over wide areas. Likewise, since potential attractants are not necessarily tree-based, attractants released by related longhorned beetles might also be of interest.*”

Because of this added paragraph, we have also changed the heading “Conclusions” for “Conclusions and future prospects”.

Line 240 “In 2018, two surveys were conducted under permissions granted by the City Council, checking all public trees for beetle infestation.” **Done!**

Line 262 “bark injuries (BI), noting also their heights above the ground and their compass orientations as one of 8 directions: N, NE, E, SE, S, SW, W, or NW. **Done!**

Line 267, delete “To set . . .” **Done!**

Line 287 “Because these 107 trees, among the 438 checked in June 2018, were still present in December, an analysis of the treatment results was included in the study. **Done!**

Line 294 “were 2-3 drillings per tree.” **Done!**

Line 311 “crown base, a non-parametric paired, 2-sample Wilcoxon Test was used that explicitly” **Done!**

Line 315 delete “here” **Done!**

Line 316 “A P-value below 0.05 leads to rejection of the null hypothesis of no difference between crown base and trunk damage.” **Done!**

Line 319 “A statistical analysis of new infestations in December 2018 was conducted in relation to past” **Done!**

Line 320 “Because the dependent variables are count data with many zeros” **Done!**

Line 332 “A tree was considered to be infested when” **Done!**

Line 415” Regression analysis was used to test if female beetles prefer to lay eggs in trees of specific heights. Tree height was not found to be a significant factor affecting the distribution of December damage compared to June (see below). Females of this beetle species are good flyers . . .” **Done!**

Line 429 “Perimeter lengths were grouped into five ranges “**Done!**

Line 443 “where the tree perimeter length had a “**Done!**

Line 458 “This result does not have an obvious explanation” **Done!**

Line 464 “Comparisons also were made of all damages” **Done!**

Line 479 “As mentioned above, all 438 trees were in this analysis, including the December 2018 data and the data contributed by the 30 trees in June that had been removed by December.”

Line 484 “The goal was” **Done!**

Line 490 and elsewhere, including line 496 “emergency” should be “emergence” **Done!**

Line 506 The compass distribution of EH (Fig 11) indicates that females prefer to lay eggs on the SW side of the tree, both on the trunk and crown base. The 150-200-cm band and the 200-250-cm band” have higher values than the others. A somewhat similar pattern emerges in the distribution of BI” **Done!**

Line 511 and elsewhere “popular” or “unpopular” should be replaced by “less preferred” or by a statement based on preference rather than popularity. Unfortunately, there are many words like this in the manuscript that should be replaced by more scientifically descriptive terminology or it will reflect negatively on the authors and the editor.

**Authors’ response:** We have addressed this point wherever necessary and believe now it reads much better.

Line 525 Do not start a new paragraph here. **Done!**

Line 526 new paragraph at “However, comparing the distribution... “**Done!**

Line 529 “This suggests that females prefer” **Done!**

Line 532 and elsewhere, “Wilcox” should be “Wilcoxon” **Done!**

Line 535 delete “Econometric” **Done!**

Line 537 “The differences between December and June in the number of new tree damages…  was compared for height and perimeter, as well as the “

**Authors’ response:** The suggested sentence does not reflect exactly what we meant since here we did not compare differences between June and December but carried out a NBRM regression analysis. Therefore, we have modified and simplified the sentence as follows (which we believe it reflects exactly what we did): “*By using* *a NBRM regression analysis, the number of new tree damages in December was explained by the damage the trees showed in June, their height and perimeter, as well as the abamectin treatment 107 trees received on April 24, 2018.*”

Line 539 “In addition the results were tested for the sum of BI +GS new damage.” **Done!**

Line 572 “The results indicate that the number of GS in June is a significant predictor of “**Done!**

Line 587 “This may be interpreted “. A statement like this is definitely discussion rather than results. While the editor considers this a plausible interpretation, there is not information about effects of chemical or visual stimuli to confirm the interpretation. It seems best to separate Results and Discussion, and then put statements like this only in the discussion. There are several other places also where it would benefit the manuscript to separate results from discussion.

**Authors’ response:** We understand the Editor’s point. However, in this paper we believe it is better to use the option “Results and discussion” instead of the option “Results” and then “Discussion”. This is because this is a rather complex study, where we are dealing with at least six groups of results (*Infestation of public mulberry trees from 2016 to 2018; *Infestation with respect to trunk height; *Infestation with respect to trunk perimeter; *Orientation and location of infestation damages; *Analysis of new infestation in December 2018 as a function of past infestation and other tree variables; *Effects of abamectin treatment), and to our understanding confining specific discussion (if needed) within each specific topic adds clarity to the paper.

Concerning our statement that “This may be interpreted as beetles reducing infestation of a severely infested tree and possibly moving to a different one.“, it is true that we have no information about effects of chemical or visual stimuli to confirm the interpretation. Although this topic is undoubtedly very interesting and we might look into it in the future, for the time being we could not say much more than what is said in this short statement. But we have changed it as follows: “*This may be interpreted as beetles reducing infestation of a severely infested tree and possibly moving to a different one, although to confirm it new research is needed about the effects of chemical and visual stimuli.*”

Line 595 “Therefore, abamectin treatment was included as a predictor in models on the full sample and a robustness analysis was included, in which a propensity score matched treated trees against nearby untreated trees with respect to the five variables listed in Table 4.” ? The original sentence was not so clear. **Done!**

Line 598 “As a result . . .” This sentence probably now should be deleted.

**Authors’ response:** No, we should keep the sentence, because here we explain that we obtained a subsample of 198 trees. Thus, it contains new information. Keep in mind that a total of 107 trees were treated with abamectin, from which only 99 were selected.

Line 601 Delete “As we see,” Then say: “After matching of propensity scores, “**Done!**

Line 602 “become close to” should be “approach” **Done!**

Line 603 “further illustrate this, not just for the means, but for the entire” **Done!**

Line 619 “the effects of abamectin were statistically significant, reducing the numbers of new infestations” **Done!**

Line 627 “The larval distributions were mapped with respect to tree height . . .”

**Authors’ response:** We have changed and simplified the original paragraph as follows:

“*We have evaluated how the infestation progressed as a function of past infestation and provided new relevant insights into female beetle’s preferences to lay their eggs on specific trees or specific areas of trees,* *hence mapping the larval distributions in them. This mapping was done by locating the infestation damages observed on the trees (their height and compass orientation), therefore providing key information to surveyors and managers on how to deal with this pest.*”
